# eHealth Literacy: Essential Skills for Consumer Health in a Networked World

**DOI:** 10.2196/jmir.8.2.e9

**Published:** 2006-06-16

**Authors:** Cameron D Norman, Harvey A Skinner

**Affiliations:** ^2^Department of Public Health SciencesUniversity of TorontoTorontoONCanada; ^1^Centre for Clinical Epidemiology & EvaluationVancouver Coastal Health Research Institute and Department of Health Care & EpidemiologyUniversity of British ColumbiaVancouverBCCanada; and Centre for Global eHealth InnovationUniversity Health NetworkUniversity of TorontoTorontoONCanada

**Keywords:** Internet, literacy, public health, health care, electronic health information, evaluation of electronic resources, electronics, telecommunications, consumer health information, patient education, educational status, computer network

## Abstract

Electronic health tools provide little value if the intended users lack the skills to effectively engage them. With nearly half the adult population in the United States and Canada having literacy levels below what is needed to fully engage in an information-rich society, the implications for using information technology to promote health and aid in health care, or for eHealth, are considerable. Engaging with eHealth requires a skill set, or *literacy,* of its own. The concept of eHealth literacy is introduced and defined as the ability to seek, find, understand, and appraise health information from electronic sources and apply the knowledge gained to addressing or solving a health problem. In this paper, a model of eHealth literacy is introduced, comprised of multiple literacy types, including an outline of a set of fundamental skills consumers require to derive direct benefits from eHealth. A profile of each literacy type with examples of the problems patient-clients might present is provided along with a resource list to aid health practitioners in supporting literacy improvement with their patient-clients across each domain. Facets of the model are illustrated through a set of clinical cases to demonstrate how health practitioners can address eHealth literacy issues in clinical or public health practice. Potential future applications of the model are discussed.

## Introduction

### Access Barriers to eHealth

What if we created tools to promote health and deliver health care that were inaccessible to over half of the population they were intended for? Consumer-directed eHealth resources, from online interventions to informational websites, require the ability to read text, use information technology, and appraise the content of these tools to make health decisions. Yet, even in countries with high rates of absolute access to the Internet, such as the United States and Canada, over 40% of adults have basic (or prose) literacy levels below that which is needed to optimally participate in civil society [1,2]. A multi-country study of information technology use and literacy found that as literacy skill levels rise, the perceived usefulness of computers, diversity and intensity of Internet use, and use of computers for task-oriented purposes rise with it, even when factors such as age, income, and education levels are taken into account [3]. If eHealth is to realize its potential for improving the health of the public, the gap between what is provided and what people can access must be acknowledged and remedied.

Greater emphasis on the active and informed consumer in health and health care [4] in recent years has led to the realization that ensuring the public has both access to and adequate comprehension of health information is both a problem [5] and an achievable goal for health services [2,3]. A recent report from the US Institute of Medicine (IOM) entitled *Health Literacy: A Prescription to End Confusion* looked at the relationship between health and literacy and found that those with limited literacy skills have less knowledge of disease management and health promoting behaviors, report poorer health status, and are less likely to use preventive services than those with average or above average literacy skills [6].

### Health Literacy

The IOM report focuses largely on health literacy, using the following definition (originally proposed by Ratzan and Parker [7]): “the degree to which individuals have the capacity to obtain, process, and understand basic health information and services needed to make appropriate health decisions” [7].

This definition underscores the importance of contextual factors that mediate health information and the need to consider health literacy in relation to the medium by which health resources are presented. Within a modern health information environment, this context includes the following: interactive behavior change tools, informational websites, and telephone-assisted services, which are all being deployed globally to promote health and deliver health care (eg, [8-[11]). However, even among North American adolescents, the highest Internet-use population in the world, many teens report that they lack the skills to adequately engage online health resources effectively [12]. There is a gap between the electronic health resources available and consumers’ skills for using them. By identifying and understanding this skill set we can better address the context of eHealth service delivery [13].

As we witness the impact that basic literacy has on health outcomes, questions arise about how literacy affects eHealth-related outcomes and experiences [14]. But unlike literacy in the context of paper-based resources, the concept of literacy and health in electronic environments is much less defined. Consumer eHealth requires basic reading and writing skills, working knowledge of computers, a basic understanding of science, and an appreciation of the social context that mediates how online health information is produced, transmitted, and received—or what can be called *eHealth literacy*. A definition and model of eHealth literacy is proposed below that describes the skills required to support full engagement with eHealth resources aimed at supporting population health and patient care.

## eHealth Literacy Model

### The Lily Model

**Figure 1:**
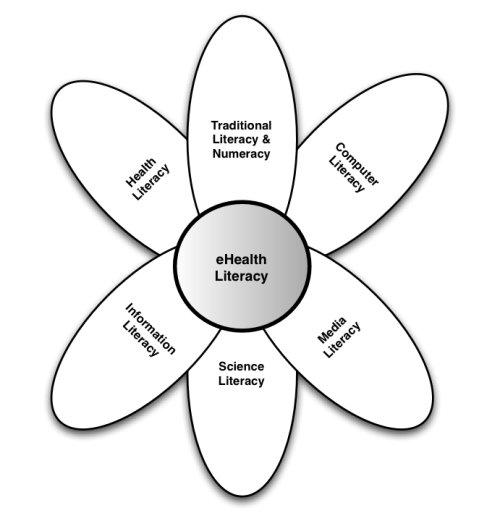
eHealth literacy lily model

Eng (2001) defines eHealth as “the use of emerging information and communication technology, especially the Internet, to improve or enable health and health care [15]; this is one of many published definitions currently in use [16]. Taken in the context of the IOM’s definition of health literacy stated above, the concept of eHealth literacy is proposed. Specifically, eHealth literacy is defined as the ability to seek, find, understand, and appraise health information from electronic sources and apply the knowledge gained to addressing or solving a health problem. Unlike other distinct forms of literacy, eHealth literacy combines facets of different literacy skills and applies them to eHealth promotion and care. At its heart are six core skills (or literacies): traditional literacy, health literacy, information literacy, scientific literacy, media literacy, and computer literacy. The relationship of these individual skills to each other is depicted in Figure 1. Using the metaphor of a lily, the petals (literacies) feed the pistil (eHealth literacy), and yet the pistil overlaps the petals, tying them together.

**Figure 2:**
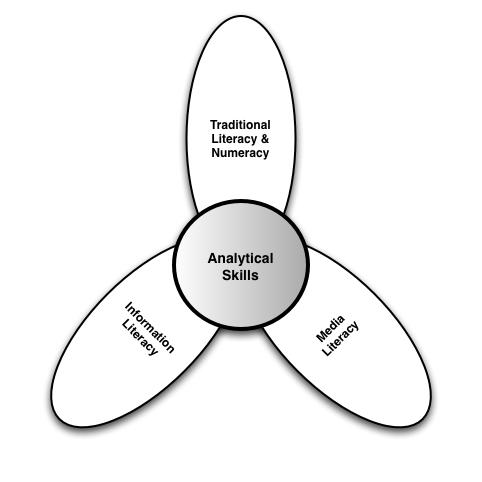
eHealth literacy analytic model

**Figure 3:**
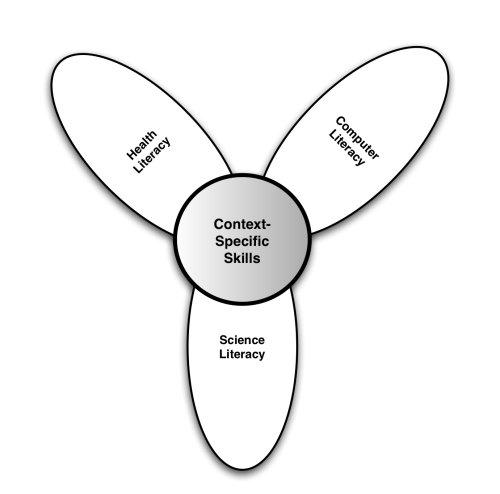
eHealth literacy context-specific model

Within the lily model, the six literacies are organized into two central types: *analytic* (traditional, media, information) and *context-specific* (computer, scientific, health). The analytic component involves skills that are applicable to a broad range of information sources irrespective of the topic or context (Figure 2), while the context-specific component (Figure 3) relies on more situation-specific skills. For example, analytic skills can be applied as much to shopping or researching a term paper as they can to health. Context-specific skills are just as important; however, their application is more likely to be contextualized within a specific problem domain or circumstance. Thus, computer literacy is dependent upon what type of computer is used, its operating system, as well as its intended application. Scientific literacy is applied to problems where research-related information is presented, just as health literacy is contextualized to health issues as opposed to shopping for a new television set. Yet, both analytic and context-specific skills are required to fully engage with electronic health resources.

eHealth literacy is influenced by a person’s presenting health issue, educational background, health status at the time of the eHealth encounter, motivation for seeking the information, and the technologies used. Like other literacies, eHealth literacy is not static; rather, it is a process-oriented skill that evolves over time as new technologies are introduced and the personal, social, and environmental contexts change. Like other literacy types, eHealth literacy is a discursive practice that endeavors to uncover the ways in which meaning is produced and inherently organizes ways of thinking and acting [17,18]. It aims to empower individuals and enable them to fully participate in health decisions informed by eHealth resources.

The six components of the eHealth literacy model are briefly outlined below.

### Traditional Literacy

This concept is most familiar to the public and encompasses basic (or prose) literacy skills such as the ability to read text, understand written passages, and speak and write a language coherently[19]. Technologies such as the World Wide Web are still text dominant, despite the potential use of sound and visual images on websites. Basic reading and writing skills are essential in order to make meaning from text-laden resources. A related issue is language itself. Over 65% of the World Wide Web’s content is in English[20], meaning that English-speakers are more likely to find an eHealth resource that is understandable and meets their needs.

### Information Literacy

The American Library Association suggests that an information literate person knows “how knowledge is organized, how to find information, and how to use information in such a way that others can learn from them” [21]. Like other literacies, this definition must be considered within the context of the social processes involved in information production, not just its application [19]. An information literate person knows what potential resources to consult to find information on a specific topic, can develop appropriate search strategies, and can filter results to extract relevant knowledge. If one views the Web as a library, with search tools (eg, Google) and a catalogue of over eight billion resources, the need for Web users to know how to develop and execute search strategies as well as comprehend how this knowledge is organized becomes imperative.

### Media Literacy

The wide proliferation of available media sources has spawned an entire field of research in the area of media literacy and media studies. Media literacy is a means of critically thinking about media content and is defined as a process to “develop metacognitive reflective strategies by means of study” [22] about media content and context. Media literacy is a skill that enables people to place information in a social and political context and to consider issues such as the marketplace, audience relations, and how media forms in themselves shape the message that gets conveyed. This skill is generally viewed as a combination of cognitive processes and critical thinking skills applied to media and the messages that media deliver [23].

### Health Literacy

As discussed earlier, health literacy pertains to the skills required to interact with the health system and engage in appropriate self-care. The American Medical Association considers a health literate person as having “a constellation of skills, including theability to perform basic reading and numerical tasks required to function in the health care environment. Patients with adequate health literacy can read, understand, and act on health care information” [24]. Consumers need to understand relevant health terms and place health information into the appropriate context in order to make appropriate health decisions. Without such skills, a person may have difficulty following directions or engaging appropriate self-care activities as needed.

### Computer Literacy

Computer literacy is the ability to use computers to solve problems[25]. Given the relative ubiquity of computers in our society, it is often assumed that people know how to use them. Yet, computer literacy is nearly impossible without quality access to computers and current information technology. For example, it is not helpful to learn PC-based commands on a Mac, to learn Windows 98 if one requires Windows XP, or be trained on a laptop when a personal digital assistant (PDA) is required for a task. Computer literacy includes the ability to adapt to new technologies and software and includes both absolute and relative access to eHealth resources. To illustrate this, Skinner and colleagues found that while nearly every Canadian teenager has access to the Internet, far fewer have the quality of access or the ability to fully utilize it for health [26,27].

### Scientific Literacy

This is broadly conceived as an understanding of the nature, aims, methods, application, limitations, and politics of creating knowledge in a systematic manner [28]. The latter-mentioned political and sociological aspects of science are in response to earlier conceptions of science as a value-free enterprise, a position that has been vigorously challenged [28-30]. For those who do not have the educational experience of exposure to scientific thought, understanding science-based online health information may present a formidable challenge. Science literacy places health research findings in appropriate context, allowing consumers to understand how science is done, the largely incremental process of discovery, and the limitations—and opportunities—that research can present.

### The Six Literacy Types

Taken together, these six literacy types combine to form the foundational skills required to fully optimize consumers’ experiences with eHealth. A profile of each literacy type with examples of the problems patient-clients might present is summarized in Table 1. Also included is a list of resources, many of them Web-based, that can be consulted to help health practitioners support patient-clients in improving their literacy skills across each domain. Although it would not be unexpected to find that older adults and those from nonindustrialized countries report greater difficulties in certain domains, particularly those that are context-specific, it is the authors’ experience that few assumptions about which groups or individuals are likely to encounter difficulties can be made. As work with highly Internet-connected populations (like North American adolescents) shows, many of whom we would expect to be skilled users, there is a lack of skills, opportunity, and environments to use eHealth to its fullest potential [12,26,27].

**Table 1:** Profile of literacy skills as related to health care practice

	**Identifying Problems**	**Potential Resources**
**Analytic**	Analytic literacy skills can be generically applied to a number of sources and circumstances. These are foundational skills that are required to participate in daily informational life. Training aids are commonly found in many countries.	
Traditional Literacy and Numeracy	Inability to read simple language Difficulty understanding printed materials in day-to-day interactions (eg, street signs) Inability to perform basic mathematical functions such as addition, subtraction, division, and multiplication with small whole numbers Difficulty in balancing a check book or calculating bank balances Difficulty reading maps or understanding simple charts	Many countries have national organizations that can provide free services for learners and professionals. Some examples include the following: Frontier College (Canada) [31] the National Literacy Trust (UK) [32] National Research and Development Centre for Adult Literacy and Numeracy (UK) [33] National Center on Adult Literacy (US) [34]
Media Literacy	Lack of awareness of media bias or perspective Inability to discern both explicit and implicit meaning from media messages Difficulty in deriving meaning from media messages	The Office of Communications strategy for enhancing media literacy (UK) [35] The National Institute of Adult Continuing Education (NIACE) media literacy guide (UK) [36] The Media Awareness Network (Canada) [37]
Information Literacy	Inability to see connections between information from various sources such as books, pamphlets, or Internet websites Lack of familiarity with libraries and other information repositories available in the community Inability to frame search questions in a manner that produces desired answers	The American Library Association has a resource page including toolkits and reference sources to aid in instruction and research [38]. Local libraries can provide support for information searches and self-directed learning; details are available through The Chartered Institute of Library and Information (UK) [39].
**Context-Specific**	Context-specific literacy skills are centered on specific issues, problem types, and contexts. These skills often require more specialized training than analytical literacy skills. Finding local resources may require more focused searches.	
Computer Literacy	Unfamiliarity with basic computer terms such as *email, mouse, keyboard,* and so forth Inability to use a mouse or other input devices Lack of exposure to computers in everyday life	Computer training courses are widespread; however, accessibility is an issue for those on fixed incomes. Many libraries offer special programs to teach patrons both computer and search skills for little or no cost. Some countries have job training centers that provide basic computer courses as part of their core mandate.
Science Literacy	Lack of understanding about the cumulative impact of scientific knowledge No awareness that science can be understood by nonscientists Unfamiliarity with science terms, the process of discovery, or the application of scientific discoveries to everyday life	Few widespread resources exist to teach people science literacy. The most common approach to learning about science is through formal education; however, many science institutions such as universities and colleges have open lectures and educational events for the public on a regular basis. In Canada, the Royal Institute for the Advancement of Science holds monthly lectures on science topics to educate the public, as does the Royal Society in the UK.
Health Literacy	Difficulty following simple self-care directions or prescription instructions Fear of taking medications without assistance Unfamiliarity or lack of understanding of basic health care terms	Two instruments have been developed and validated for use in assessing health literacy: The Test of Functional Health Literacy in Adults (TOFHLA) and the Rapid Estimate of Adult Literacy in Medicine (REALM). Both have been widely used and are designed to assess health literacy within 30 minutes. The TOFHLA has 67 items and includes a numeracy component (the ability to read and understand numbers) and a reading comprehension component. The REALM has 66 items. Fostering health literacy is a challenge; however, attending to people’s media preferences (text, video, audio) and using plain language in interactions is a place to begin.

These six skill types illustrate the challenges that eHealth presents to those with low literacy in any one area. Although one need not have mastery in all these areas to benefit from eHealth resources, it can be argued that without moderate skills *across* these literacies, effective eHealth engagement will be unlikely. Using a specific health-related issue (smoking prevention and cessation) as an example, Table 2 illustrates how these literacy issues may present within the context of primary care while suggesting possible intervention strategies. Unlike other areas of health care, there is no “best practice” solution to addressing problems of literacy that fits into a single session or neatly packaged brief intervention. Rather, improving literacy is a process that requires coordinated remediation and education, involving partnerships among patient-clients, practitioners, educators, and community health organizations over time. It is as much a process as it is an outcome.

**Table 2:** Case scenarios: tobacco use and the six literacy types

**Case Study**	**Literacy Type(s) Required**
A group practice has decided to provide smoking prevention resources for teens and their parents on its website. The resources are to be approved by a patient advisory committee. The three sites put forward are Phillip Morris USA’s smoking prevention material site [40], The Smoking Zine by TeenNet at the University of Toronto [41], and Health Canada’s Quit4Life program [42].	**Media Literacy:** Teens need to know the difference between the perspectives presented on each site to make an informed decision. One site belongs to a tobacco company with a vested interest in selling cigarettes, and it advocates prevention strategies not supported by the best evidence. The other two sites are from a teen-focused research project at a public university and from a government health agency. These three sites together encourage discussion about media issues and allow for exploration with patient-clients the ways in which information on one issue can be presented differently. The Media Awareness Network [37] has resources for working with children and youth in enhancing media literacy that can aid in fostering this discussion.
A 60-year-old man with little formal education and no experience using computers presents with concerns about continuing to smoke. He has made many unsuccessful quit attempts and has been told there are Internet resources available that can help him. He is interested in trying something different to help him stop using tobacco.	**Traditional Literacy:** A basic literacy assessment should be undertaken before recommending use of the Internet as a resource. This may be done by having the patient read a few simple text passages from consumer health materials or the newspaper or by asking the patient directly if he has difficulties reading. If basic text materials are difficult, the person is likely to require assistance in using the Web or other Internet resources even at a rudimentary level. **Computer Literacy:** If the man has limited experience with computers, specific training through a local library, community center, or other community program might be necessary to provide him with the means to use Web-assisted tobacco interventions. This requires that the practitioner arrange and assist the patient in connecting with one of these community resources or inquire if there are family members or friends who can assist him in getting online.
A 35-year-old woman presents with an interest in finding information on smoking to share with her teenage daughter. She uses email at work and regularly visits a local website for news, but otherwise does not surf regularly and does not know how to find Internet resources easily.	**Information Literacy:** A referral to the local library or on-staff librarian (if available) is the simplest strategy. A short tutorial on the use of search engines, search strategies, and health databases can provide the basics on how to navigate the Internet for health information. Once basic search strategies have been established, the patient may wish to use evidence-supported resources for evaluating consumer health information, available through tools such as the DISCERN Project websites [43,44].
A 24-year-old mother of two small children and current smoker challenges the claim that second- hand smoke is harmful to her children, citing research she found on the Internet.	**Science Literacy:** This scenario presents a teachable moment to outline some of the issues that address science literacy, such as how evidence changes over time and issues of quality. In this case, it may be useful to direct the patient to reference sources outlining contrary views and encourage a dialogue around what makes good science. It is possible the research she has referred to is out of date, contested, or heavily biased (eg, tobacco-industry sponsored).
A 45-year-old patient has been prescribed nicotine replacement therapy (NRT) using an inhaler. The patient is unsure when to use the inhaler and under what conditions and reports behaviors that indicate he is not using the inhaler as originally prescribed.	**Health Literacy:** The presenting patient is following the product instructions. It is worth exploring the context around this behavior to see if it is a matter of fit between the NRT delivery method and the person or whether it is an issue of literacy. Patient instructions should be reviewed to ensure that they are written in plain language. Practitioners may also wish to explore whether there are other media tools available from the manufacturer or local health unit that can be used to supplement the written instructions, such as visual aids or videos to reduce the amount of required reading.

## Discussion

Literacy is as much a process as an outcome and requires constant attention and upgrading. The key is to reach a level of fluency at which one can achieve working knowledge of the particular language (or skill), enough to function at a level conducive to achieving health goals. Knowledge, information, and media forms are context-specific, and context dictates what skills and skill levels are required to access health resources. For example, technical jargon may be appropriate in academic discourse provided it allows for a more precise explanation of certain concepts. However, when directed at nontechnical consumers or those outside of a particular research or practice culture, technical language may need to undergo a translation process in order to convey a message properly[45]. Whereas a scientist may be interested in acetylsalicylic acid, a patient requiring pain relief knows this substance only as Aspirin or ASA.

As the World Wide Web and other technology-based applications become a regular part of the public health and health care environment, viewing these tools in light of the skills required for people to engage them becomes essential if the power of information technology is to be leveraged to promote health and deliver health care effectively. The eHealth literacy model presented here is the first step in understanding what these skills are and how they relate to the use of information technology as a tool for health. The next step is to apply this model to everyday conditions of eHealth use—patient care, preventive medicine and health promotion, population-level health communication campaigns, and aiding health professionals in their work—and evaluate its applicability to consumer health informatics in general. Using this model, evaluation tools can be created and systems designed to ensure that there is a fit between eHealth technologies and the skills of intended users. By considering these fundamental skills, we open opportunities to create more relevant, user-friendly, and effective health resources to promote eHealth for all.
